# 1-[(4-{[(2-Oxo-1,2-dihydro­naphthalen-1-yl­idene)meth­yl]amino}­anilino)methyl­idene]naphthalen-2(1*H*)-one dihydrate

**DOI:** 10.1107/S1600536811041870

**Published:** 2011-10-12

**Authors:** Anita Blagus, Branko Kaitner

**Affiliations:** a Department of Chemistry, J.J. Strossmayer University, Osijek, Franje Kuhača 20, HR-31000 Osijek, Croatia; b Laboratory of General and Inorganic Chemistry, Department of Chemistry, Faculty of Science, University of Zagreb, Horvatovac 102a, HR-10002 Zagreb, Croatia

## Abstract

The title compound, C_28_H_20_N_2_O_2_·2H_2_O, comprises a Schiff base mol­ecule with an imposed inversion centre in the middle of *p*-phenyl­enediamine unit and water mol­ecules of crystallization. In the structure, the Schiff base mol­ecule is present as the keto–amino tautomer with a strong intra­molecular N—H⋯O hydrogen bond. The Schiff base mol­ecules and water mol­ecules of crystallization create infinite [010] columns through O—H⋯O hydrogen bonds. Inter­molecular attractions within columns are through additional π–π inter­actions [centroid–centroid distance = 3.352 (1) Å] between parallel Schiff base mol­ecules. The columns are joined into infinite (011) layers through weak C—H⋯O hydrogen bonds. The layers pack in an assembly by van der Waals attractions, only being effective between bordering non-polar naphthalene ring systems.

## Related literature

For general background to Schiff bases, see: Blagus *et al.* (2010 ▶). The stereochemistry of intrinsic Schiff bases differs significantly, see: Inabe *et al.* (1994 ▶). For the quinoid effect in 2-oxy-naphthaldimine Schiff base derivatives, see: Gavranić *et al.* (1996 ▶); Friščić *et al.* (1998 ▶). For the herringbone packing motif in fused aromatic systems, see: Desiraju & Gavezzotti (1989 ▶).
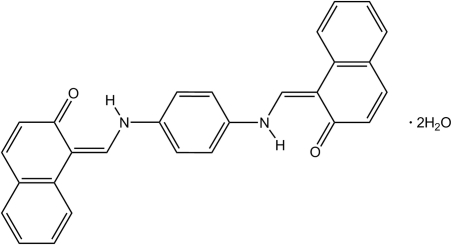


## Experimental

### 

#### Crystal data

C_28_H_20_N_2_O_2_·2H_2_O
*M* *_r_* = 452.49
Monoclinic, 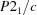

*a* = 17.4222 (11) Å
*b* = 4.4686 (5) Å
*c* = 15.9374 (10) Å
β = 116.30 (1)°
*V* = 1112.3 (2) Å^3^
*Z* = 2
Mo *K*α radiation
μ = 0.09 mm^−1^
*T* = 298 K
0.5 × 0.2 × 0.1 mm

#### Data collection

Oxford Diffraction Xcalibur CCD diffractometer
14006 measured reflections
2423 independent reflections
1351 reflections with *I* > 2σ(*I*)
*R*_int_ = 0.054

#### Refinement

*R*[*F* ^2^ > 2σ(*F* ^2^)] = 0.061
*wR*(*F* ^2^) = 0.190
*S* = 1.05
2423 reflections
160 parameters
3 restraints
H atoms treated by a mixture of independent and constrained refinement
Δρ_max_ = 0.20 e Å^−3^
Δρ_min_ = −0.27 e Å^−3^

### 

Data collection: *CrysAlis CCD* (Oxford Diffraction, 2003 ▶); cell refinement: *CrysAlis CCD*; data reduction: *CrysAlis RED* (Oxford Diffraction, 2003 ▶); program(s) used to solve structure: *SHELXS97* (Sheldrick, 2008 ▶); program(s) used to refine structure: *SHELXL97* (Sheldrick, 2008 ▶); molecular graphics: *ORTEP-3* (Farrugia, 1997 ▶); software used to prepare material for publication: *WinGX* (Farrugia, 1999 ▶), *PARST97* (Nardelli, 1995 ▶) and *Mercury* (Macrae *et al.*, 2006 ▶).

## Supplementary Material

Crystal structure: contains datablock(s) I, global. DOI: 10.1107/S1600536811041870/kp2357sup1.cif

Structure factors: contains datablock(s) I. DOI: 10.1107/S1600536811041870/kp2357Isup2.hkl

Supplementary material file. DOI: 10.1107/S1600536811041870/kp2357Isup3.cml

Additional supplementary materials:  crystallographic information; 3D view; checkCIF report

## Figures and Tables

**Table 1:** Hydrogen-bond geometry (Å, °)

*D*—H⋯*A*	*D*—H	H⋯*A*	*D*⋯*A*	*D*—H⋯*A*
N1—H1⋯O1	0.86	1.86	2.560 (3)	138
O1*W*—H1*B*⋯O1	0.83	2.27	3.090 (4)	169
O1*W*—H1*A*⋯O1^i^	0.84	2.01	2.826 (4)	165
C13—H13⋯O1*W*^ii^	0.93	2.33	3.247 (5)	170

Symmetry codes: (i) 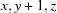; (ii) 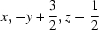.

**Fig. 1.**
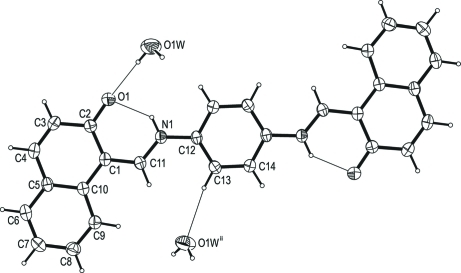
A general overview of (I) showing numbering scheme with anisotropic thermal ellipsoids pictured at 30% probability level. Thin lines display intra N–H···O, and the two intermolecular hydrogen bonds O–H···O and C–H···O. Hydrogen atoms are drawn as spheres of arbitrary radius.

**Fig. 2.**
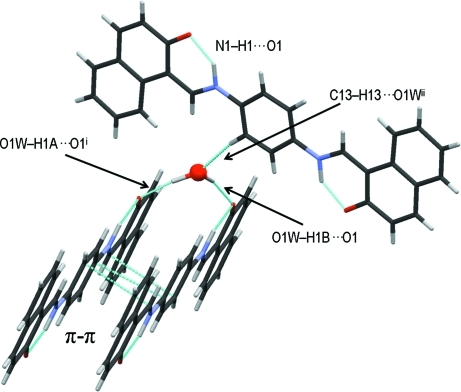
The display of three intermolecular contacts of water molecule with nearby Schiff base molecules. Two Schiff base molecules connected by π–π interactions are additionally bridged with a water molecule through two strong hydrogen bonds O1W–H1A···O1i [(i): x, y + 1, z] and O1W–H1B···O1, respectively. The third intermolecular contact of water molecule is via C13–H13···OW1ii 3.247 (5) Å [(ii): x, - y + 3/2, z] interaction.

**Fig. 3.**
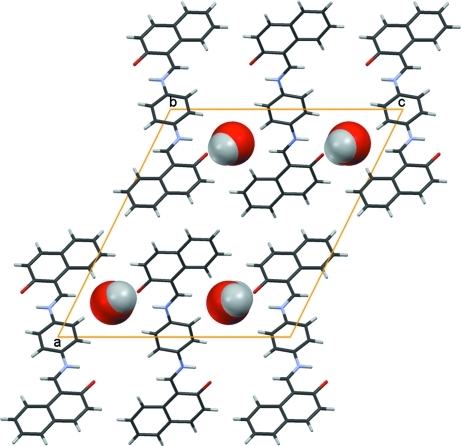
Water molecules of crystallisation occupy the cylindrical voids formed by assembling Schiff base molecules into the [010] columns.

**Fig. 4.**
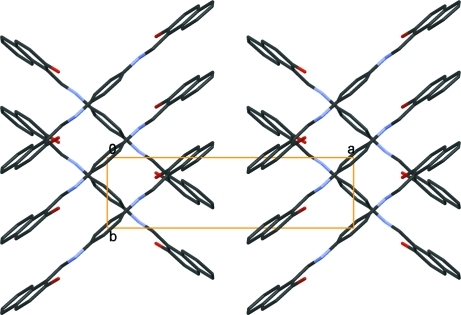
The display of herringbone packing arrangement of Schiff base molecules viewed down c-axes. Water molecules and all hydrogen atoms were omitted for clarity.

## supplementary crystallographic information

### Comment

Desiraju and Gavezzotti presented classification of packing arrangements for polynuclear aromatic hydrocarbons depending on the number and positions of C and H atoms in molecules (Desiraju & Gavezzotti, 1989). As a result of significant planarity, Schiff bases derived from *p-*phenylenediamine have significant aromatic-aromatic C···C interactions compared to the number of intermolecular C–H hydrogen bonds and usually show a herringbone motif of intermolecular assembly (Blagus *et al.*, 2010).

We report here the crystal structure of the title compound (I) as a crystal hydrate. The stereochemistry of intrinsic 1,4-bis(2-hydroxy-1-naphthylmethylideneamino)benzene was earlier determined and reported (Inabe *et al.*, 1994). Distinctively planar Schiff base molecule possesses crystallographic inversion centre in the middle of *p*-phenylenediamine moiety with *anti* arrangements of chelate rings (Fig. 1). Interplanar angle between naphthalene moiety and the central aromatic ring is 1.7 (2)°. Schiff base molecule does not deviate significantly from planarity in contrast to the structure by Inabe *et al.*  with corresponding interplanar angle being 22.3°.In both structures molecules possess internal (molecular) symmetry with inversion centre in the middle of *p*-phenylenediamine moiety.

Bond distances C2–O1 [1.277 (4) Å] and C11–N1 [1.322 (4) Å] indicate keto-amino tautomeric form of (I). This is confirmed by a formation of strong intramolecular hydrogen bond N–H···O [N···O = 2.560 (3) Å]. Short C3-to-C4 bond distance [1.345 (5) Å] with O1 oxygen atom at C2 position of naphthalene core indicates the presence of quinoid effect (Gavranić *et al.*, 1996; Friščić *et al.*, 1998). Water molecules play crucial role in crystal packing: a) as bridging media pilling up Schiff base molecules at the separation characteristic for graphite in the form of infinite [010] columns and b) as bridging molecule connecting neighboring [010] columns into infinite (011) layers. Hydrogen bonds effective for columns formation are: a) O1W–H1A···O1^i^ 2.826 (4) Å [(i): *x*, *y* + 1, *z*] and O1W–H1B···O1 3.090 (4) Å while for layers formation is effective b) C13–H13···OW1^ii^ 3.247 (5) Å [(ii): *x*, – *y* + 3/2, *z*] (Fig. 2 and Table 1).

Along with intermolecular contacts *via* hydrogen bonds the linking between molecules amplifies through  π–π interactions with offset (Fig. 2). Schiff base molecules arrange parallel to each other with their middle *p*-phenylenediamine moiety being separated at distances characteristic for layer separation in graphite. The shortest separations corresponding to the sum of van der Waals radii are: C12···C12^iii^ 3.362 (3) Å [(iii): -*x*, -*y* + 1, -*z*] and C11···C13^iv^ 3.374 (3) Å [(iv): *x*, *y* - 1, *z*]. π··· π interactions are also characterised by perpendicular *Cg*^iii^···*Cg*^v^ distance 3.352 (1) Å [(v): -*x*, 3 - *y*, -*z*] and slippage of 2.955 Å. There is a space between each pair of neighboring columns large enough to accommodate water molecules (Fig. 3). Connection between neighboring parallel layers is accomplished through bordering non-polar naphthalene core by the standard van der Walls attractions. The rather planar Schiff base molecules of (I) reveal characteristic herringbone motif of packing arrangement (Fig. 4).

### Experimental

The crystals of (I), (1,4-bis(2-hydroxy-1-naphthylmethylideneamino)benzene as crystal hydrate were obtained during an unsuccessful attempt to synthesise the nickel complex of corresponding Schiff base. Schiff base itself was prepared separately in standard way by condensation of 2-hydroxy-naphtaldehyde and *p*-phenylenediamine in ethanol solution in molar ratio 2:1 and used as a ligand in metal complex synthesis. The 1:1 mixture of 0.1 mmol DMSO solutions of Schiff base and 0.2 mmol nickel salt, NiCl_2_. 6H_2_O was stirred under reflux for two h at 373 K. Preparation of nickel complex failed. The crystals of title compound crystallised from mother liquor after cooling to RT and mechanically separated from nickel salt.

### Refinement

Hydrogen atoms were refined in two different ways.

For hydrogen atoms bonded to C and N atoms benzene type riding mode was used with C-to-H and N-to-H bond distances taken as 0.93 and 0.86 Å, respectively.

Due to somewhat higher values of anisotropic thermal parameters of O1W oxygen atom, implying to certain disorder of water molecule, bond distances O1W to H1A and H1B, respectively, as well as bond distance H1A–H1B were restrained to the values accepted for water molecule. Bond distances O1W to H1A and H1B, respectively were fixed to 0.82 (1) Å and H1A to H1B to 1.30 (1) Å and the position of hydrogen atoms were re-calculated in consecutive refinement cycles. Isotropic thermal parameters for hydrogen atoms were estimated as 1.2 times of equivalent isotropic thermal parameter of corresponding C, N and O atoms.

### Crystal data

**Table:**

C_28_H_20_N_2_O_2_·2H_2_O	*F*(000) = 476
*M**_r_* = 452.49	*D*_x_ = 1.351 Mg m^−^^3^
Monoclinic, *P*2_1_/*c*	Mo *K*α radiation, λ = 0.71073 Å
Hall symbol: -P 2ybc	Cell parameters from 2423 reflections
*a* = 17.4222 (11) Å	θ = 4–27°
*b* = 4.4686 (5) Å	µ = 0.09 mm^−^^1^
*c* = 15.9374 (10) Å	*T* = 298 K
β = 116.30 (1)°	Prism, green
*V* = 1112.3 (2) Å^3^	0.5 × 0.2 × 0.1 mm
*Z* = 2	

### Data collection

**Table:**

Oxford Diffraction Xcalibur CCD diffractometer	1351 reflections with *I* > 2σ(*I*)
Radiation source: fine-focus sealed tube	*R*_int_ = 0.054
graphite	θ_max_ = 27.0°, θ_min_ = 3.9°
ω scans	*h* = −22→21
14006 measured reflections	*k* = −5→5
2423 independent reflections	*l* = −20→20

### Refinement

**Table:**

Refinement on *F*^2^	Primary atom site location: structure-invariant direct methods
Least-squares matrix: full	Secondary atom site location: difference Fourier map
*R*[*F*^2^ > 2σ(*F*^2^)] = 0.061	Hydrogen site location: inferred from neighbouring sites
*wR*(*F*^2^) = 0.190	H atoms treated by a mixture of independent and constrained refinement
*S* = 1.05	*w* = 1/[σ^2^(*F*_o_^2^) + (0.0914*P*)^2^ + 0.2128*P*] where *P* = (*F*_o_^2^ + 2*F*_c_^2^)/3
2423 reflections	(Δ/σ)_max_ = 0.009
160 parameters	Δρ_max_ = 0.20 e Å^−^^3^
3 restraints	Δρ_min_ = −0.27 e Å^−^^3^

### Special details

**Table:**

Geometry. All e.s.d.'s (except the e.s.d. in the dihedral angle between two l.s. planes) are estimated using the full covariance matrix. The cell e.s.d.'s are taken into account individually in the estimation of e.s.d.'s in distances, angles and torsion angles; correlations between e.s.d.'s in cell parameters are only used when they are defined by crystal symmetry. An approximate (isotropic) treatment of cell e.s.d.'s is used for estimating e.s.d.'s involving l.s. planes.
Refinement. Refinement of *F*^2^ against ALL reflections. The weighted *R*-factor *wR* and goodness of fit *S* are based on *F*^2^, conventional *R*-factors *R* are based on *F*, with *F* set to zero for negative *F*^2^. The threshold expression of *F*^2^ > σ(*F*^2^) is used only for calculating *R*-factors(gt) *etc*. and is not relevant to the choice of reflections for refinement. *R*-factors based on *F*^2^ are statistically about twice as large as those based on *F*, and *R*- factors based on ALL data will be even larger.

### Fractional atomic coordinates and isotropic or equivalent isotropic displacement parameters (Å^2^)

**Table:**

	*x*	*y*	*z*	*U*_iso_*/*U*_eq_	
O1	0.19806 (12)	0.2820 (4)	0.25053 (12)	0.0537 (6)	
N1	0.12888 (13)	0.5855 (4)	0.09888 (14)	0.0420 (5)	
H1	0.1321	0.5349	0.1524	0.050*	
C1	0.24546 (15)	0.2518 (5)	0.13184 (17)	0.0383 (6)	
C2	0.25000 (16)	0.1743 (5)	0.22164 (17)	0.0416 (6)	
C3	0.31492 (17)	−0.0329 (6)	0.27967 (18)	0.0486 (7)	
H3	0.3186	−0.0853	0.3378	0.058*	
C4	0.37040 (18)	−0.1525 (6)	0.25139 (19)	0.0495 (7)	
H4	0.4116	−0.2855	0.2910	0.059*	
C5	0.36876 (15)	−0.0839 (5)	0.16289 (18)	0.0427 (6)	
C6	0.42800 (18)	−0.2111 (6)	0.1358 (2)	0.0527 (7)	
H6	0.4696	−0.3410	0.1764	0.063*	
C7	0.42563 (19)	−0.1479 (7)	0.0517 (2)	0.0583 (8)	
H7	0.4654	−0.2326	0.0345	0.070*	
C8	0.36356 (19)	0.0436 (6)	−0.0084 (2)	0.0568 (8)	
H8	0.3616	0.0850	−0.0665	0.068*	
C9	0.30524 (18)	0.1733 (6)	0.01493 (19)	0.0498 (7)	
H9	0.2643	0.3014	−0.0274	0.060*	
C10	0.30542 (15)	0.1174 (5)	0.10235 (17)	0.0387 (6)	
C11	0.18466 (15)	0.4573 (5)	0.07490 (17)	0.0399 (6)	
H11	0.1832	0.5066	0.0175	0.048*	
C12	0.06465 (15)	0.7953 (5)	0.04716 (18)	0.0396 (6)	
C13	0.05037 (17)	0.8940 (6)	−0.04079 (19)	0.0466 (7)	
H13	0.0838	0.8227	−0.0685	0.056*	
C14	−0.01402 (16)	1.0993 (6)	−0.08723 (18)	0.0450 (6)	
H14	−0.0233	1.1673	−0.1461	0.054*	
O1W	0.1500 (2)	0.8211 (6)	0.3392 (2)	0.0919 (9)	
H1A	0.160 (3)	0.978 (4)	0.317 (3)	0.110*	
H1B	0.170 (3)	0.687 (5)	0.318 (3)	0.110*	

### Atomic displacement parameters (Å^2^)

**Table:**

	*U*^11^	*U*^22^	*U*^33^	*U*^12^	*U*^13^	*U*^23^
O1	0.0561 (12)	0.0580 (12)	0.0539 (12)	0.0074 (10)	0.0306 (10)	0.0081 (9)
N1	0.0396 (12)	0.0385 (11)	0.0463 (12)	0.0040 (10)	0.0177 (10)	0.0037 (10)
C1	0.0330 (13)	0.0333 (12)	0.0435 (14)	−0.0026 (11)	0.0122 (11)	−0.0004 (11)
C2	0.0396 (14)	0.0392 (14)	0.0462 (15)	−0.0029 (12)	0.0193 (13)	0.0003 (12)
C3	0.0490 (16)	0.0481 (15)	0.0434 (15)	0.0008 (13)	0.0156 (13)	0.0080 (12)
C4	0.0414 (15)	0.0442 (15)	0.0511 (16)	0.0031 (13)	0.0097 (13)	0.0079 (13)
C5	0.0323 (13)	0.0402 (13)	0.0495 (15)	−0.0007 (11)	0.0124 (12)	−0.0009 (12)
C6	0.0394 (15)	0.0461 (16)	0.0653 (19)	0.0066 (12)	0.0166 (14)	0.0016 (14)
C7	0.0535 (18)	0.0555 (17)	0.074 (2)	0.0034 (15)	0.0361 (17)	−0.0068 (16)
C8	0.0609 (19)	0.0574 (18)	0.0573 (18)	0.0021 (15)	0.0309 (16)	−0.0027 (14)
C9	0.0511 (17)	0.0498 (16)	0.0480 (16)	0.0090 (13)	0.0214 (14)	0.0045 (12)
C10	0.0345 (13)	0.0333 (12)	0.0442 (14)	−0.0025 (11)	0.0138 (11)	−0.0025 (11)
C11	0.0373 (14)	0.0352 (13)	0.0449 (14)	−0.0005 (11)	0.0162 (12)	−0.0029 (11)
C12	0.0365 (14)	0.0328 (13)	0.0451 (14)	0.0008 (11)	0.0142 (12)	−0.0017 (11)
C13	0.0433 (15)	0.0454 (14)	0.0536 (16)	0.0058 (13)	0.0238 (13)	0.0002 (13)
C14	0.0454 (15)	0.0457 (14)	0.0427 (14)	0.0052 (13)	0.0185 (13)	0.0039 (12)
O1W	0.113 (2)	0.0952 (18)	0.0958 (19)	0.0004 (18)	0.0719 (17)	0.0129 (17)

### Geometric parameters (Å, °)

**Table:**

O1—C2	1.277 (3)	C6—H6	0.9300
N1—C11	1.322 (3)	C7—C8	1.378 (4)
N1—C12	1.412 (3)	C7—H7	0.9300
N1—H1	0.8600	C8—C9	1.357 (4)
C1—C11	1.392 (3)	C8—H8	0.9300
C1—C2	1.440 (3)	C9—C10	1.414 (4)
C1—C10	1.452 (3)	C9—H9	0.9300
C2—C3	1.437 (3)	C11—H11	0.9300
C3—C4	1.345 (4)	C12—C13	1.383 (4)
C3—H3	0.9300	C13—C14	1.383 (4)
C4—C5	1.431 (4)	C13—H13	0.9300
C4—H4	0.9300	C14—H14	0.9300
C5—C6	1.404 (4)	O1W—H1A	0.836 (10)
C5—C10	1.419 (3)	O1W—H1B	0.831 (10)
C6—C7	1.353 (4)		
			
C11—N1—C12	127.8 (2)	C6—C7—C8	119.2 (3)
C11—N1—H1	116.1	C6—C7—H7	120.4
C12—N1—H1	116.1	C8—C7—H7	120.4
C11—C1—C2	119.8 (2)	C9—C8—C7	121.9 (3)
C11—C1—C10	120.7 (2)	C9—C8—H8	119.1
C2—C1—C10	119.5 (2)	C7—C8—H8	119.1
O1—C2—C3	119.5 (2)	C8—C9—C10	121.3 (3)
O1—C2—C1	122.1 (2)	C8—C9—H9	119.4
C3—C2—C1	118.4 (2)	C10—C9—H9	119.4
C4—C3—C2	121.2 (2)	C9—C10—C5	116.3 (2)
C4—C3—H3	119.4	C9—C10—C1	123.9 (2)
C2—C3—H3	119.4	C5—C10—C1	119.8 (2)
C3—C4—C5	122.8 (2)	N1—C11—C1	122.8 (2)
C3—C4—H4	118.6	N1—C11—H11	118.6
C5—C4—H4	118.6	C1—C11—H11	118.6
C6—C5—C10	120.3 (2)	C13—C12—N1	122.9 (2)
C6—C5—C4	121.3 (2)	C12—C13—C14	119.5 (2)
C10—C5—C4	118.4 (2)	C12—C13—H13	120.2
C7—C6—C5	121.1 (3)	C14—C13—H13	120.2
C7—C6—H6	119.4	C13—C14—H14	119.6
C5—C6—H6	119.4	H1A—O1W—H1B	103 (2)
			
C11—C1—C2—O1	2.0 (4)	C8—C9—C10—C1	179.3 (2)
C10—C1—C2—O1	−178.9 (2)	C6—C5—C10—C9	1.3 (4)
C11—C1—C2—C3	−178.5 (2)	C4—C5—C10—C9	−178.7 (2)
C10—C1—C2—C3	0.6 (3)	C6—C5—C10—C1	−178.9 (2)
O1—C2—C3—C4	179.6 (2)	C4—C5—C10—C1	1.1 (3)
C1—C2—C3—C4	0.0 (4)	C11—C1—C10—C9	−2.3 (4)
C2—C3—C4—C5	−0.1 (4)	C2—C1—C10—C9	178.6 (2)
C3—C4—C5—C6	179.6 (3)	C11—C1—C10—C5	177.9 (2)
C3—C4—C5—C10	−0.5 (4)	C2—C1—C10—C5	−1.2 (3)
C10—C5—C6—C7	−0.7 (4)	C12—N1—C11—C1	−179.9 (2)
C4—C5—C6—C7	179.3 (3)	C2—C1—C11—N1	−0.9 (3)
C5—C6—C7—C8	−0.3 (4)	C10—C1—C11—N1	−179.9 (2)
C6—C7—C8—C9	0.7 (4)	C11—N1—C12—C13	1.3 (4)
C7—C8—C9—C10	−0.1 (4)	N1—C12—C13—C14	179.8 (2)
C8—C9—C10—C5	−0.9 (4)		

### Hydrogen-bond geometry (Å, °)

**Table:**

*D*—H···*A*	*D*—H	H···*A*	*D*···*A*	*D*—H···*A*
N1—H1···O1	0.86	1.86	2.560 (3)	138
O1W—H1B···O1	0.83	2.27	3.090 (4)	169
O1W—H1A···O1^i^	0.84	2.01	2.826 (4)	165
C13—H13···O1W^ii^	0.93	2.33	3.247 (5)	170

Symmetry codes: (i) *x*, *y*+1, *z*; (ii) *x*, −*y*+3/2, *z*−1/2.
